# Structural Features of the Mental Foramen in a Saudi Subpopulation: A Retrospective CBCT Study

**DOI:** 10.1155/2021/1138675

**Published:** 2021-12-10

**Authors:** Mohammed Mashyakhy, Ahmed Mostafa, Amani Abeery, Zainab Sairafi, Nazeeha Hakami, Riyadh Alroomy, Hitesh Chohan, Abdulaziz Abu-Melha

**Affiliations:** ^1^Department of Restorative Dental Sciences, College of Dentistry, Jazan University, Jazan, Saudi Arabia; ^2^Department of Oral Maxillofacial and Diagnostic Sciences, College of Dentistry, Jazan University, Jazan, Saudi Arabia; ^3^General Dental Practitioner, Jazan, Saudi Arabia; ^4^Department of Restorative Dental Sciences, College of Dentistry, Majmaah University, Al Majmaah, Saudi Arabia; ^5^Department of Restorative Dental Sciences, College of Dentistry, King Khalid University, Abha, Saudi Arabia

## Abstract

**Introduction:**

Accurate and precise knowledge about the position, size, and shape of the mental foramen (MF) are critical in avoiding procedural complications. The MF's anatomical features vary among different ethnic groups, and various radiographic techniques have been used to determine these variations.

**Aims:**

To evaluate the MF's shape, vertical and horizontal positions, and distance from the border of the mandible. To evaluate the differences among genders as they pertain to the right and left sides of the mandible and research the bilateral symmetry regarding the same variables.

**Materials and Methods:**

Cone beam computer tomography (CBCT) scans of 155 Saudi patients (69 males and 86 females) who visited the college of dentistry's clinics were obtained from the college database for this retrospective study. All the scans were analyzed by 3 calibrated examiners. The data collected was analyzed statistically, and results were obtained.

**Results:**

The MF was located under the mandibular second premolar in 56.9% of cases, whereas in 26.9% of cases, it was located between the first and second mandibular premolar. The most prevalent position was below the level of the apices of the mandibular premolar teeth (87.2%). The round shape was most frequent (44.9%) compared to the H-oval (34.7%) and V-oval (20.4%). The V-oval shape was more frequent in males, while the H-oval shape was more frequent in females. The average distance from the center of the MF to the mandibular border was 14.03 ± 1.58 mm, with males exhibiting a greater distance than females. Overall, there were no significant differences between the bilateral symmetry and the right and left sides for all parameters.

**Conclusion:**

The most common position of the MF was under the root apex of the mandibular second premolar, with an average distance of about 14 mm from the border of the mandible. The position and shape of the MF were the same bilaterally in the majority of individuals.

## 1. Introduction

The mental foramen (MF) is a highly important anatomical landmark in the mandible that can be defined as a bilateral funnel-like opening on the buccal surface of the mandible. It is mostly presented inferiorly and between the root tips of the mandibular premolars. The mental nerve, a branch of the inferior alveolar nerve adjunct to blood vessels, exits through the MF [1–3]. The mental nerve innervates the lower lip, mentum, labial mucosa, lower incisors, canines, and premolars [4, 5]. MF is an essential landmark for diagnostic and clinical procedures. A precise knowledge of its position, shape, and size is crucial for successful and complication-free dental procedures such as surgical implant placement, endodontic surgeries, and any osteotomies in the region [6]. The importance of locating the MF is not limited to the dental field; plastic surgeons and emergency physicians for procedures that include periapical surgery, orthognathic surgery, the repair of lower lip and chin lacerations, and facial reconstructive surgery also need to know the MF's position [7, 8]. Paresthesia is a serious complication when neurovascular bundles of mental nerves get damaged during a dental procedure in the area [9].

The position of the MF can vary among different populations [10]. Various techniques have been utilized to determine the position of MF, including cadaveric dissection, panoramic radiographs, periapical radiographs, magnetic resonance imaging, computed tomography, and cone-beam computed tomography (CBCT) [11]. Most of these techniques have limitations such as cost, radiation exposure, and magnification. CBCT is the most recent and accurate in vivo modality that provides a clear and precise position of the MF before the dental procedure [12]. CBCT is not only effective in detecting MF but it is also effective in detecting the presence of accessory MF, which has been determined to exist in 6.4% of the Australian population [13]. It has been reported that a patient had a total of five accessory MF (three in the right side and two in the left side) [14]. In contrast, a patient was reported to have no MF on the right side and hypoplastic MF on the left side. Interestingly, the patient reported no sensory disturbances [15]. Upon literature search, only four studies have reported the MF in a Saudi population (drawn from the country's central, northern, and eastern regions) using CBCT [16–19]. These studies covered a few structural features of MF, such as the position of MF, course of mental nerve (MN), and distance to adjacent cortical plate with sample sizes ranging from 94 to 302 CBCT scans.

Therefore, this study is aimed at evaluating the vertical and horizontal position and shape of the MF and its distance from the mandible body. In addition, gender differences and bilateral symmetry were evaluated using the same variables in a Saudi subpopulation drawn from the country's southern region.

## 2. Material and Methods

### 2.1. Sample Selection

In the present radiographic retrospective study, radiographs from 155 Saudi national patients were collected by CBCT between 2017 and 2019. Of the 155 patients, the 69 male patients and 86 female patients had a mean age of 28.74 ± 9.56 years. These radiographs were obtained from the databank archives of the College of Dentistry, Jazan University at the city of Jazan in the southern region of Saudi Arabia, where the scans were used for different diagnostic purposes. All CBCT scans were collected retrospectively; patients were not exposed to X-ray for this study. An ethical approval given by the Institute's Ethical Committee was obtained before commencing the study (Reference#: CODJU-21024). In this study, a total of 254 right and left sides of clear images of the MF were studied, on the basis of the following criteria: the presence of permanent dentition, and those teeth should be adjacent to the MF from the canine to the first molar of one or both sides of the mandible. The exclusion criteria were as follows: distorted/unclear cone-cut CBCT images, the presence of mixed dentition, and the presence of any lesion obscuring the MF region.

### 2.2. Radiographic Evaluation

The CBCT machine, 3D Accuitomo 170 (MORITA, Japan) with 90 Kv, 5–8 mA, 17.5 s exposure time, and 0.25 mm voxel size, was used. Morita's i-Dixel 3D software imaging program was used for processing the CBCT radiographs. Panoramic, axial, coronal, and sagittal radiographical segments were acquired for the MF area. In this study, the horizontal and vertical position, shape, and distance to the mandible border of the MF were evaluated as the main outcome. Comparisons among gender, differences between the right and left sides, and bilateral symmetry were evaluated as secondary outcomes.

**Figure 1:**
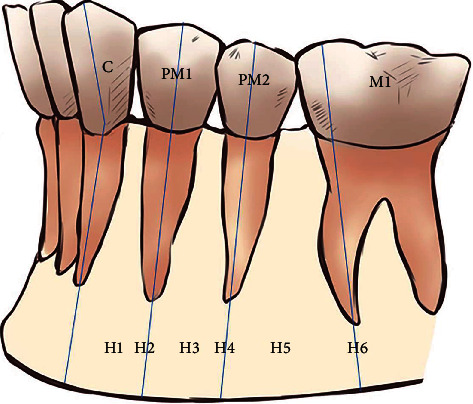
Schematic representation of the horizontal (anterior–posterior) position of the mental foramen in relation to the lower teeth (C: canine; PM1: first premolar; PM2: second premolar; M1: first molar). Position (H1) between the C and PM1, (H2) in line with the long axis of the PM1, (H3) between the long axes of the PM1 and PM2, (H4) in line with the long axis of the PM2, (H5) between the long axes of the PM2 and M1, and (H6) in line with the long axis of the mesial root of the first lower molar.

The Tebo and Telford classification was used to establish the following horizontal relationships of MF to the mandibular teeth (Figure 1) [20]:

(H1) the MF is between the canine and first premolar

(H2) the MF is at the level of the first premolar

(H3) the MF is between the first and second premolars

(H4) the MF is at the level of the second premolar

(H5) the MF is between the second premolar and the first molar

(H6) the MF is at the level of the first molar

**Figure 2:**
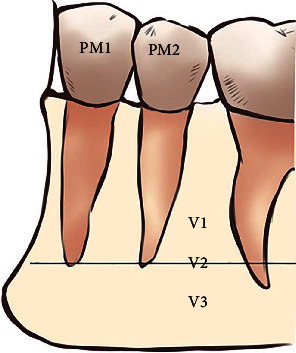
Vertical (superior–inferior) position of the mental foramen in relation to the apices of the mandibular premolar teeth (PM1: first premolar; PM2: second premolar). Position (V1) above the level of the apices of the PM1 and PM2, (V2) at the level of the apices of the PM1 and PM2, and (V3) below the level of the apices of the PM1 and PM2.

The vertical position of the MF according to the root apices of the lower premolars was classified into three types (Figure 2) [21, 22]:

(V1) the MF was located above the level of the apices of the first and second mandibular premolar teeth

(V2) the MF was located at the level of the apices of the first and second mandibular premolar teeth

(V3) the MF was located below the level of the apices of the first and second mandibular premolar teeth

The shape of the MF was categorized into three types after measuring the horizontal and vertical dimension of the MF [22]: (a) oval horizontal (H-oval) when *H* : *V* was over 1.24, (b) oval vertical (V-oval) when *H* : *V* was less than 0.76, and (c) round: when 0.76 ≤ *H* : *V* ≥ 1.24.

**Figure 3:**
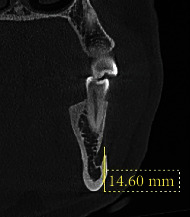
CBCT coronal view shows the distance from the middle of the MF to the border of the mandible.

The distance from the MF to the border of the mandible was calculated from a coronal view by measuring the distance from the center of the foramen to the border of the mandible (Figure 3).

The scans were evaluated by two calibrated dentists with 2 years of experience in reading CBCT scans for different diagnostic purposes. In the absence of a consensus, an endodontist with more than 8 years of experience in reading CBCT scans finalized the decision.

### 2.3. Statistical Analysis

Data was recorded in a master sheet (Microsoft Excel 2016), coded, and double-checked. First, a normality test was used to explore the distribution of the data, which revealed nonnormal distributions. Accordingly, nonparametric tests were used. The results were presented as frequencies and percentages for the different positions and shapes and as means and standard deviations for the distance of the MF to the border. Distribution by gender (regardless of side) and by side (regardless of gender) of the different positions and shapes was examined using the Chi-squared test. However, the differences in the distance were tested using the Mann–Whitney *U* test. For bilateral symmetry, subjects with complete parameters on the right and left sides were included. Cohen's Kappa test was used to measure the symmetry of the different positions and shapes, whereas the Wilcoxon Signed Rank Test was used to evaluate the differences in distance. The statistical software program SPSS v.26 for Windows (IBM Corp., Armonk, NY, USA) was used for analysis, with *P* value < 0.05 as the significance level.

## 3. Results

The study comprised 155 subjects (having one or both MFs), with a total of 274 MFs screened for position and shape. However, the mandibular border was not clear on 20 sides, leading to the inclusion of 254 MFs for distance measurement. For bilateral symmetry, only subjects with both MFs were included (*N* = 110).

The overall distribution of the different *H*- and *V*-positions and shapes is presented in Table 1. H1 was not detected in the surveyed subjects. The most frequent H-position was H4 (56.9%), followed by H3 (29.9%), whereas the least frequent was H6 (2.2%). For the V-position, the most frequent was V3 (87.2%), followed by V2 (9.1%) and then V1 (3.6%). Regarding the MF shape, the round shape was the most frequent (44.9%), followed by H-oval (34.7%) and then V-oval (20.4%).

**Figure 4:**
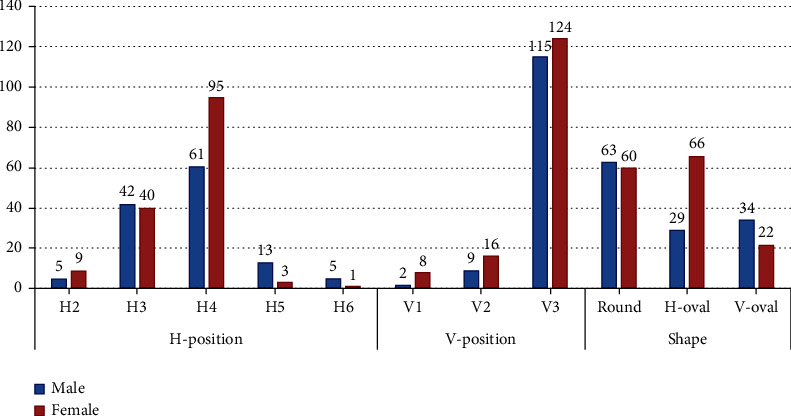
Distribution of the different positions and shapes of the MF by gender.

Figure 4 shows the distribution of the different H- and V-positions and shapes of the MF based on gender. A significant difference was observed in the distribution of the H-position (*P* = 0.003). H6 was more frequent in males, followed by H5, whereas H2 was more frequent in females, followed by H4. No significant difference was observed in the distribution of the V-position among males and females (*P* = 0.125). However, a highly significant difference was found in the distribution of the MF shape (*P* < 0.001). V-oval was more frequent in males, whereas H-oval was more frequent in females.

**Figure 5:**
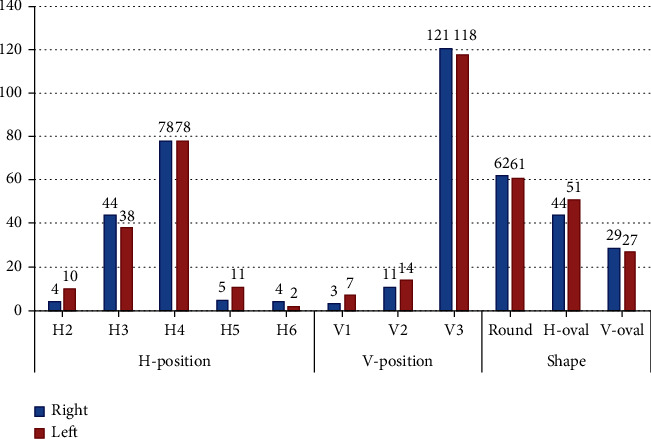
Distribution of the different positions and shapes of the MF by sides.

The differences in the distribution of H- and V-positions and shapes by side are shown in Figure 5. In general, no significant differences were observed between the right and left sides for all parameters (*P* > 0.05).

The distance from the center of the MF to the mandibular border is presented in Table 2. The overall distance was 14.03 ± 1.58 mm, ranging from 10.92 mm to 19 mm. The difference between males and females was highly significant (*P* < 0.001), with males having a higher distance than females (14.87 ± 1.43 mm vs. 13.31 ± 1.33 mm). However, no significant difference was observed between the right and left sides (*P* = 0.626).

The bilateral symmetry of the H-position was highly significant (*P* < 0.001). Of the 110 subjects, 66 (60%) had the same H-position of the MF on both sides (Table 3). Similarly, the symmetry of the V-position was highly significant (*P* < 0.001). About 91 (83%) subjects exhibited the same V-position of the MF on both sides (Table 4). In addition, the symmetry of the MF shape was highly significant (*P* < 0.001). A total of 62 (56%) subjects had the same shape of the MF on both sides (Table 5). Regarding bilateral symmetry of the distance to the mandibular border, no significant difference was found between right and left distances among the subjects (right side = 14.11 ± 1.55 mm, left side = 13.93 ± 1.51 mm; *P* = 0.201).

## 4. Discussion

Identifying the vital structures around the root apex of a tooth scheduled for endodontic surgery, such as the MF position, is critical for effective treatment. In comparison of two-dimensional imaging techniques, CBCT is a superior instrument for linear measurements [23]. Previous studies in the Saudi population reported only three variables including the position of MF, distance to adjacent cortical plate, and the course of the MN. Beside the position of MF, the present study evaluated two other very important variables: the shape of MF and its distance to the border of the mandible, both of which are important when planning a surgery in the area [6].

The present study reported the MF was most prominent in H4 and V3, which is at the level of the mandibular second premolar horizontally and below its apex vertically. With regard to gender distribution, only the H-position differed significantly between males and females. The MF was more prevalent in H6 and H5 among males. However, it was more prevalent in H2 and H4 among females. Only the horizontal and vertical oval shapes of the MF differed significantly between males and females. The V-shape was more common among males, whereas the H-shape was more frequent among females. In addition, the distance from the center of the MF to the border of the mandible was higher in males than in females. The vertical and horizontal positions of the MF and its shape were in the same position and had the same shape bilaterally, in the majority of the individuals in our research. In the present study, there was no accessory, absence, or hypoplastic MF in any of the CBCT images studied. These variations are quite unusual and present a limitation of our study. They may necessitate a larger sample size.

In the Kingdom of Saudi Arabia, four previous studies investigated the position of the MF using CBCT. Al-Mahalawy et al. [16] found that the position of the MF in 52.8% of the subjects in the eastern region of the Kingdom was below the mandibular second premolar, whereas that of 29.6% of the subjects was between the premolars. Furthermore, Aldosimani et al. [17] reported that the position of the MF in more than 68.1% of the subjects in the central region of the Kingdom was in close proximity to the mandibular second premolar. Additionally, Mahabob et al. [19] and Srivastava [18] demonstrated that MFs are located below second premolars in eastern and northern regions, respectively. Those studies were in agreement with the findings of our study. Moreover, the same studies observed a significant difference between males and females in the vertical position of the MF, which was more apical to the premolar apices in males compared with females, and they hypothesized that the difference could be due to the difference in jaw size between males and females. Al-Mahalawy et al. [16] and our study, however, found no significant difference, even though both have a large sample size.

The position of the MF varies among ethnic groups [24]. Our findings were similar to those of Alam et al. [25], who investigated the Saudi, Egyptian, and Jordanian populations and concluded that the majority of them presented the MF under the long axis of the second mandibular premolar. However, other studies found that the most prevalent position of the MF was between the premolars [26–29].

The study measured the distance from the inferior border of the mandible to the middle of the MF because the crestal bone can be impacted by resorption, resulting in length variation from the upper border of the mandible to the middle of the MF. Therefore, a clinician should be aware of such measurements when performing genioplasty or apical curettage in the premolar area. We found that the overall distance was 14.03 ± 1.58 mm. This result was almost consistent with that of Al-Mahalawy et al. [16], who reported an average distance of 13.8 mm. This small discrepancy was attributed to the measuring point, which, in Al-Mahalawy et al. began from the inferior margin of the MF, whereas we began from the center of the MF. Notably, we detected a statistically significant difference between males and females, although Al-Mahalawy et al. [16] observed no significant difference. This finding may be due to the small sample size, as gender variations require a large sample size to be explored precisely. These measurements were consistent with those of von Arx et al. [26] and Kalender et al. [30], who reported average distances of 13.2 and 12.4 mm.

Regarding the MF's shape, the round shape was more prevalent, which was consistent with Alam et al. who investigated Jordanian and Egyptian populations. By contrast, the oval shape was more prevalent among Sri Lankans, Peruvians, and North Indians [31–33].

The current study has some limitations, including its small sample size; however, the sample size was higher than some studies in the same population. Furthermore, the present study examined other variables (shape and distance from border of the mandible) not previously studied in earlier studies in the Saudi population. Although our small sample suggests that the result cannot be generalized to the entire population, it is interesting nonetheless and adds to the body of knowledge in the Saudi population.

Another limitation was that we did not investigate whether our participants received orthodontic treatment or had skeletal malocclusion, which might have influenced the results.

Also, the influence of missing teeth around the MFs was not evaluated and compared to edentulous individuals in regards to position, shape, and distance to the border of the mandible. In addition, the CBCT scan was evaluated by calibrated dentists and an endodontist. No dento-maxillofacial radiologist was included, and the evaluation was concluded by consensus and not done independently. In future research, we suggest utilizing comprehensive variables to explore the MF's position in different subpopulations in the Kingdom. Furthermore, we suggest measuring the upper border of the mandible using the cementoenamel junction of the teeth as a measurement point, because crestal bone might be impacted by resorption [34].

## 5. Conclusion

Within the limitations of our study, the MF was most prevalent below the level of the second mandibular premolar. The MF in males was highly prevalent in H6 and H5. However, it was more common in H2 and H4 among females. The V-shape was more prevalent in males, whereas the H-shape was more common in females. In addition, males exhibited a greater distance from the center of the MF to the border of the mandible than females. The vertical and horizontal positions of the MF and its shape were in the same positions, and shaped bilaterally, in the majority of the subjects in our study.

## Figures and Tables

**Table 1:** Overall distribution of the different positions and shapes of right and left MF (*N* = 274).

	*N*	%
Horizontal position		
H2	14	5.1
H3	82	29.9
H4	156	56.9
H5	16	5.8
H6	6	2.2
Vertical position		
V1	10	3.6
V2	25	9.1
V3	239	87.2
Shape		
Round	123	44.9
H-oval	95	34.7
V-oval	56	20.4

**Table 2:** Distance of MF to the mandibular border for overall and according to gender and side (*N* = 254).

Overall	14.03 ± 1.58		
By gender	Male	Female	
	14.87 ± 1.43	13.31 ± 1.33	*P* < 0.001

By side	Right	Left	
	14.09 ± 1.63	13.97 ± 1.53	*P* = 0.626

**Table 3:** Bilateral symmetry of horizontal position of MF among participants (*N* = 110).

		Left side					
		H2	H3	H4	H5	H6	Total
Right side	H2	2	2	0	0	0	4
	H3	4	18	14	2	0	38
	H4	3	11	44	4	0	62
	H5	0	1	1	1	0	3
	H6	0	0	0	2	1	3
	Total	9	32	59	9	1	110

Kappa test; *P* < 0.001.

**Table 4:** Bilateral symmetry of vertical position of MF among participants (*N* = 110).

		Left side			
		V1	V2	V3	Total
Right side	V1	2	0	1	3
	V2	3	2	4	9
	V3	2	9	87	98
	Total	7	11	92	110

Kappa test; *P* < 0.001.

**Table 5:** Bilateral symmetry of MF shape among participants (*N* = 110).

		Left side			
		Round	H-oval	V-oval	Total
Right side	Round	29	17	5	51
	H-oval	13	21	3	37
	V-oval	4	6	12	22
	Total	46	44	20	110

Kappa test; *P* < 0.001.

## Data Availability

The data supporting the findings of this research are available from the corresponding author upon a reasonable request.
